# Endometriosis Symptomatology, Dyspareunia, and Sexual Distress Are Related to Avoidance of Sex and Negative Impacts on the Sex Lives of Women with Endometriosis

**DOI:** 10.3390/ijerph20043362

**Published:** 2023-02-14

**Authors:** Georgia Privitera, Kerry O’Brien, RoseAnne Misajon, Chung-Ying Lin

**Affiliations:** 1 School of Social Sciences, Faculty of Arts, Monash University, Wellington Road, Clayton 3800, Australia; 2 The Cairnmillar Institute, Tooronga Road, Hawthorn East 3123, Australia; 3 Institute of Allied Health Sciences, College of Medicine, National Cheng Kung University, Tainan 70101, Taiwan

**Keywords:** endometriosis, sexual intercourse, pain, sexual avoidance, dyspareunia, symptomatology, sex life, pelvic pain, women’s health, sexual distress

## Abstract

Background: Endometriosis affects approximately 10% of women and is associated with a range of symptoms including pelvic pain, abnormal bleeding, and painful sexual intercourse. However, very little is known about the relationship between endometriosis-related symptoms and sex. Methods: Women with a diagnosis of endometriosis (*n* = 2060; mean age = 30 years) completed a questionnaire measuring the frequency of endometriosis symptoms, dyspareunia, sexual distress, avoidance of sex, and the perceived negative impact of endometriosis symptoms on sex life. Results: In bivariate and multivariate logistic regression models with avoidance of sex and perceived negative impact of endometriosis symptoms on sex life as DVs, higher endometriosis symptom frequency, dyspareunia, and sexual distress were associated with greater avoidance of sex and higher perceived negative impact of endometriosis symptoms on sex life. With a two- and three-fold increase in the odds of avoiding sex and reporting a negative impact of endometriosis on sex lives, respectively, for each point increase in dyspareunia. Similarly, there was a 7% to 11% increase in avoidance of sex and the negative impact of endometriosis on sex lives, per one-point increase in symptom frequency and sexual distress. Conclusions: The results highlight the considerable impacts of endometriosis symptomatology on women’s sex lives and wellbeing. Better medical and counselling services may be needed to ameliorate the negative impact of endometriosis on women’s sex lives.

## 1. Introduction

Endometriosis is a chronic non-communicable condition affecting approximately 10% of women of reproductive age, with approximately 176 million women diagnosed worldwide [1]. It is characterized by the presence of endometrial-like tissue outside the uterus and is present in 60% to 70% of women undergoing surgery for pelvic pain symptoms [2]. Abnormally located endometrial-like tissue can lead to the development of symptoms such as pelvic pain, pain during menstruation, pelvic inflammatory disease, infertility, and pain during and after sexual intercourse [3,4]. These symptoms may significantly impact women’s sexual experiences, but there is a paucity of research examining the relationship between endometriosis-related symptomatology (specifically endometriosis symptom frequency and dyspareunia), sexual distress and perceived negative impacts of endometriosis symptoms on sex lives, and sexual avoidance. The current research seeks to address this gap in the evidence.

There are three general forms of endometriosis-related pain: dysmenorrhea, dyspareunia, and non-menstrual chronic pelvic pain [5]. Dysmenorrhea is defined as pelvic pain arising from menstrual bleeding [6], while dyspareunia is classified as pain in the genital area or within the pelvis associated with sexual intercourse [7]. Dyspareunia can be attributed to endometrial lesions which are commonly located in the posterior pelvic cavity [2]. These lesions form hard nodules and during sexual intercourse, these nodules are affected by an external impact force. The tension of contractile hard lesions, which lack elasticity, increases and shifts, resulting in deep sexual pain, which some patients may also experience after sexual intercourse [8]. Dyspareunia, in particular, appears to be associated with a range of sex-related problems including difficulties with achieving arousal and orgasm [9,10] and women with dyspareunia have been reported to avoid or limit sexual activity due to pain [11]. A systematic review of surgical interventions for endometriosis suggested that problems with achieving arousal and orgasm may be due to fear and/or anticipation of pain, known inhibitors of the sexual response cycle (Barbara et al., 2017). Moreover, these types of sexual problems are thought to have a negative impact on sexual relationships and associated wellbeing [12]. Some research has found that women who experience sexual activity-related pain report greater psychological distress, more negative attitudes towards their sexuality, and higher levels of impairment in sexual function [13].

General endometriosis-related pelvic pain has also been found to have a negative impact on women’s psychological health and wellbeing [14,15,16]. Similarly, endometriosis-related dyspareunia is associated with impairments in sexual functioning including decreased sexual activity, pre-sex anxiety and physical tension, less satisfying orgasms, intimate-partner relationship problems, and poorer sex-related quality of life [2,10,17,18]. Women with endometriosis were also found to have less sexual intercourse, or be sexually abstinent because of pain and endometriosis symptomatology, and to have poorer self-esteem than women with other serious gynecological conditions [19]. A literature review also found that a range of psychological comorbidities (e.g., depression, anxiety) accompanied experiences of dyspareunia fear and distress in women with higher levels of endometriosis-related symptomatology [20].

Despite the adverse impact of these endometriosis-related sexual problems on women’s psychological, social, and physical wellbeing and quality of life, the field has largely ignored their importance to women. Notably, the 10th World Congress on Endometriosis (2008) established priority issues for research which largely ignored the reduced quality of sex life in women with endometriosis [20]. However, there is a clear need for research that seeks to understand the relationship between endometriosis symptomatology (endometriosis symptom frequency and dyspareunia) and women’s sex-related distress and the avoidance of sex and perceived negative impacts of endometriosis symptoms on women’s sex lives. Research has found that around 50% of women with symptomatic endometriosis report that their compromised sex life has had the largest impact on their quality of life [21]. Although existing studies have provided useful insights into the impact of dyspareunia on women’s sex lives, these samples have rarely focused on women with endometriosis. Existing studies on endometriosis, although useful, have often involved small and/or select samples (i.e., women who are undergoing surgery for endometriosis or women under a certain age). Additionally, although endometriosis symptom frequency has been shown to negatively impact other areas of women’s lives [19], previous research has not directly examined the relationship between experiencing endometriosis-related symptoms and both avoidance of sex and the perceived extent of the negative impact of endometriosis symptoms on women’s sex lives. This is important in order to extend the knowledge of the impact of endometriosis on sex and to help identify potential medical and counselling needs aimed at improving sex lives and relationships. The current study intends to provide information for healthcare providers that may inform support and treatment needs for women with endometriosis. Specifically, to address the lack of research in this area this study aimed to (1) measure sexual distress, avoidance of sex and the perceived negative impact of endometriosis symptoms on women’s sex lives, and (2) examine the relationship between endometriosis symptom frequency, dyspareunia, sexual distress, and avoidance of sex (DV) and the perceived negative impact of endometriosis symptoms on women’s sex lives (DV).

## 2. Materials and Methods

### 2.1. Participants

Women with a diagnosis of endometriosis (*n* = 2060) completed an online questionnaire. The majority of the sample were surgically diagnosed (87.8%) with 12.2% diagnosed by a medical practitioner. The mean age in the sample was 30.49 years (SD = 7.25), with participants’ ages ranging from 18 to 62 years. Most participants (75.1%) were in a committed relationship and identified as heterosexual (89.6%), were sexually active (95.3%), and were engaging in sexual intercourse on average 5.52 times per month (SD = 5.81). Most of the sample (88.9%) were from Australia (*n* = 1478), Europe (*n* = 182), and New Zealand (*n* = 172). The participant characteristics are detailed in Table 1.

The present study is part of a larger study examining women’s experiences of PCOS, endometriosis, and a combination of both. More comprehensive details of the sample are available in previous research [16]. The total endometriosis sample contained 2078 participants, a small portion (*n* = 18) entered the study but did not provide answers on key demographic characteristics and measures, leaving a final sample of 2060 participants.

Participant inclusion/ exclusion criteria were as follows; participants must be aged 18 years or above, have a formal diagnosis of endometriosis, and have English language competency. For the current study, women reporting a diagnosis of both endometriosis and Polycystic Ovarian Syndrome (PCOS) were excluded from our analysis. The minimum required sample size for the larger study was calculated using the standard rule of thumb adopted in mainstream epidemiology research whereby 20 participants are required per cell and level of the variable included in the analysis (20; demographics, psychological, and social variables) [22]. Accordingly, the minimum required sample for the analysis to be conducted was, approximately, 960 women (4 × 3 × 4 × 20). Assuming incomplete data for approximately 15% (*n* = 144), the total minimum sample size required was 1104.

### 2.2. Procedure

The study was advertised via peak endometriosis organizations in Australia, New Zealand, and the United Kingdom via their social media pages (Facebook, Instagram, and Twitter) and associated word-of-mouth/snowballing. The advertisements included a link to the questionnaire hosted by the Qualtrics research platform. On average, the questionnaire took 25 min to complete. Participants were informed at the start of the study that completing the questionnaire represented their consent to participate in the study. Ethical approval was granted by the University Human Research Ethics Committee.

### 2.3. Materials

Along with demographic characteristics (i.e., age, stage of endometriosis, years with a diagnosis, and delay in diagnosis), the questionnaire assessed endometriosis symptomatology, dyspareunia, female sexual distress, avoidance of sex, and the negative impact of endometriosis symptoms on sex life.

#### 2.3.1. Endometriosis Symptomatology

To measure endometriosis symptom frequency, participants were presented with a list of common endometriosis symptoms the authors had devised after consulting various medical sources and asked to indicate which symptoms they generally experienced. Consistent with previous research [19], a symptom frequency score was then created and utilized for analysis.

#### 2.3.2. The Biberoglu and Behrman Scale (B&B)

The Pelvic Pain Subscale of the B&B scale assesses three pain symptoms; pelvic pain not related to the menses, dysmenorrhea (painful menstruation), and dyspareunia (pain experienced before, during, or after sexual intercourse). Each of the three types of pelvic pain are rated on a 4-point rating scale ranging from 0 = absent to 3 = severe, and these three items can be summed to form a single score [23]. Because the study sought to only examine the effect of dyspareunia, we only utilized the B&B item directly assessing this (B&B item 3).

#### 2.3.3. The Female Sexual Distress Scale-Revised (FSDS-R)

The FSDS-R is a screening instrument which consists of 13 items for measuring sex-related personal distress in women. The fixed-choice response format offered a five-point response scale ranging from 0 = never to 4 = always, with items summed to create a total score ranging from 0 to 52. Severe sexual distress is sometimes defined by an FSDS-R cut-off score of 11 or greater. However, for the present study, we used the total score as a linear variable [24]. Cronbach’s alpha for the scale in the present study was 0.96.

#### 2.3.4. Avoidance of Sex and Impact on Sex Life

We developed two questions assessing the impact of endometriosis on the avoidance of sex, i.e., “Do you avoid sexual intercourse because of your endometriosis?” (avoidance of sex), and on sex life, i.e., “Do your endometriosis symptoms have a negative impact on your sex life?” (perceived negative impact on sex life). Participants simply answered yes (coded as 1) or no (coded as 0) for each of the items.

### 2.4. Statistical Analysis

Spearman’s Rho was used to examine bivariate relationships between endometriosis symptom frequency, dyspareunia, sexual distress, avoidance of sex, and perceived negative impact of endometriosis symptoms on sex life. Multivariate logistic regression models were used to examine multivariate relationships between the variables of interest and the dependent variables avoidance of sex and perceived negative impact of endometriosis symptoms impact on sex life. A priori tests revealed no significant differences between heterosexual and non-heterosexual participants (≈10%) on any of the variables of interest in pre-analyses. Accordingly, we reported the combined results for heterosexual and non-heterosexual women. We reported means (M) and standard deviations (SD), proportions (%) for descriptive statistics, and r’s, beta’s (B) and standard errors (SE), adjusted odds ratios (adjORs) with 95% confidence intervals, and associated *p*-values for all statistics.

## 3. Results

### 3.1. Descriptives

Women on average experienced 10.87 (SD = 4.81) endometriosis symptoms. The mean dyspareunia (B&B) score for the sample was 1.67 (SD = 0.91), with approximately twenty per cent of women (20.4%) reporting severe dyspareunia, 35.6% reporting moderate dyspareunia, and 34.4% reporting mild dyspareunia. Only 9.6% reported no dyspareunia. Mean sexual distress (FSDS-R) scores were high (M = 25.84, SD = 13.74). Using the clinical cut-off score of ≥11, 68.3% of the sample reported having severe levels of sexual distress.

Eighty per cent (80.3%) of participants reported that they believed their endometriosis symptoms have had a negative impact on their sex life and 67.5% reported avoiding sexual intercourse due to their endometriosis.

### 3.2. Bivariate Correlations

There were significant bivariate relationships between most of the variables of interest (see Table 2). Notably, greater endometriosis symptom frequency was associated with more avoidance of sex and a higher perceived negative impact of endometriosis symptoms on sex life. More severe dyspareunia was associated with more avoidance of sex and a higher perceived negative impact of endometriosis symptoms on sex life. Higher sexual distress scores were also associated with more avoidance of sex and higher perceived negative impact of endometriosis symptoms on sex life. The largest coefficients were for relationships between dyspareunia and sexual distress (FSDS-R), and avoidance of sex and perceived negative impact of endometriosis symptoms on sex life.

### 3.3. Multivariate Logistic Regression Models

Multivariate logistic regression models found several significant relationships between the independent variables and avoidance of sex and the perceived negative impact of endometriosis symptoms on sex life (see Table 3). Age, endometriosis symptom frequency, dyspareunia, and sexual distress were all associated with greater avoidance of sex. Notably, dyspareunia had the largest association with avoidance of sex, and for each 1-point increase in dyspareunia scores (scale range 0–3), there was a 139% increase in the odds of women reporting avoiding sex. There was also a 7% and 8% increase in the odds of avoiding sex in women for each 1-point increase in symptom frequency and sexual distress, respectively. The overall model examining associations with avoidance of sex was significant (X^2^ (5, 1557) = 567.41, *p* ≤ 0.001).

For the perceived negative impact of endometriosis symptoms on women’s sex lives, endometriosis symptom frequency, dyspareunia, and sexual distress were all associated with women reporting a perceived negative impact of endometriosis symptoms on their sex life. Again, the largest association was between the level of dyspareunia and the perceived negative impact of endometriosis symptoms on sex life with a 176% increase in the odds of reporting endometriosis having a negative impact on their sex life. There were also smaller but significant increases (7% and 11%) in the odds of women reporting a negative impact of endometriosis symptoms on their sex life for each 1-point increase in symptom frequency and sexual distress, respectively. The overall model was also significant (X^2^ (5, 1554) = 567.98, *p* ≤ 0.001).

## 4. Discussion

There is a paucity of research examining the role of endometriosis symptomatology (symptom frequency and dyspareunia) and associated sexual distress on the avoidance of sex and the negative impact of endometriosis symptoms on women’s sex lives. In the present sample, eight out of ten women reported that their endometriosis symptoms had a negative impact on their sex lives, 68.3% reported experiencing severe sexual distress, and over two-thirds (67%) said they avoided sex because of their endometriosis symptomatology. In both bivariate and multivariate analyses endometriosis symptom frequency, dyspareunia, and sexual distress, were associated with more avoidance of sex and the reporting of a greater negative impact on their sex lives. The impact of dyspareunia was particularly strong and concerning, with a two- and three-fold increase in the odds of avoiding sex and reporting a negative impact on sex lives, respectively, for each point increase in dyspareunia. This points to the need for better options for the management of pain during and after sex. The overall results highlight the need for better medical and counselling options to support women and their partners in having a sex life, and importantly an enjoyable one.

Although we examined several novel relationships between endometriosis-related variables and outcomes, the general findings appear to be in line with previous research in the area. For example, previous studies have found similarly high rates of dyspareunia and sexual distress in women with endometriosis and noted the severe impairments that endometriosis causes to women’s sexual functioning [10,25]. For example, it has been reported that women experienced feelings of guilt and loss of femininity due to endometriosis-related sexual problems [10]. Other work examining the negative impact of endometriosis on women’s sex lives also mirrors the results of the present study [26,27]. A qualitative study of endometriosis found women reported avoiding sexual intercourse due to pain, which in turn led to feelings of inadequacy and guilt [26]. Similarly, quantitative research found that a large portion of women experienced problems in their sex life due to endometriosis, which also affected their intimate relationships [27]. Overall, the findings from this study combined with those detailed above highlight the extent to which sex lives are compromised for many women with endometriosis, and likely their partners.

The results are also supported by conceptual models, such as the Fear Avoidance Model (FAM) [28,29], which suggest that experiences of pain lead to avoidance of enjoyable and important behaviors, sometimes even after the pain is reduced/removed. For example, a study found that women with fears arising from chronic pelvic pain reported greater anxiety and avoidance of sex, and lower sexual activity and libido [29]. The authors suggest that pain-related fear leads to defensive behaviors and pain-related anxiety leads to avoidant behaviors and hypervigilance, placing an individual at risk of negative pain cognitions and/or behaviors [29]. Importantly, this learned fear associated with sex may persist even after pain-related symptoms have been reduced.

While large, our sample is one of convenience and caution needs to be taken in the interpretation of the results. In particular, prevalence estimates cannot and were not made, and the numbers of women reporting avoidance of sex and other scores across measures may not represent those of the theoretical population of interest. However, it is reasonable to assume the pattern of the relationships identified in this study will be similar in other samples of women with endometriosis. Additionally, the findings are consistent with the existing body of research on endometriosis, sexual concerns, and distress. The present sample was recruited online through endometriosis organizational webpages and support groups. This may have resulted in a sample with more severe endometriosis symptomology and therefore a greater need to seek out information and support. Our advertised study criteria stated we were seeking women with a formal diagnosis of endometriosis, which means that women with endometriosis-related symptoms who have not had a formal diagnosis were excluded from the sample. How this affects the results is uncertain, however, if the symptoms experienced by those without a diagnosis are similar to those reported here, there is reason to expect a similar pattern or results. Further, in this paper we report on women who have a self-reported diagnosis of endometriosis, meaning that the reported diagnosis was not accompanied by a medical report independently confirming the veracity of the self-reported diagnosis; consequently, this may result in some women who have not been formally diagnosed completing the survey, for example, women who are in the process of being diagnosed or women who believe they have endometriosis, but not clinically confirmed. However, the self-report data on endometriosis have been found to be highly accurate in studies where medical register data are not available [30]. Finally, many of the treatments provided for endometriosis may affect the sex lives of women with endometriosis in different ways. We were unable to assess this possibility in the present study, but such research is needed.

## 5. Conclusions

Notwithstanding the limitations, the present study provides preliminary evidence on the often-considerable extent of women’s sex-related problems and distress due to their endometriosis symptomatology. Better medical and counselling services may be needed to ameliorate the negative impact of endometriosis on women’s sex lives. In the absence of a cure for endometriosis, approaches for reducing and managing dyspareunia and distress are needed, if only in the form of short-term pain relief. The impact of dyspareunia on women’s sex lives not only affects their physical and mental wellbeing but impacts on intimate relationships and family. More research is needed in this understudied area. Work that seeks to understand the impact of endometriosis-related pain on partners and family is needed, as wellbeing is further compromised if personal relations suffer. Additionally, research on pain-reduction treatments that can allow women to have and enjoy sex with their partners if even for limited periods, appear to be urgently needed.

## Figures and Tables

**Table 1:** Participant Characteristics.

Characteristics (*n* = 2060)	Mean ± SD	Range
Age in years	30.49 ± 7.25	18–62
Age of diagnosis	24.65 ± 6.53	10–49
Age of onset of symptoms	16.85 ± 5.54	8–45
Delay in diagnosis (years)	7.78 ± 6.20	0–40
Years with a diagnosis	5.54 ± 5.88	0–36
Sexually active (*n* = 2054)	Frequency	Percent
Yes	1958	95.3%
No	96	4.7%
Sex per month (*n* = 1796)	Mean ± SD	Range
Sexual intercourse episodes per month	5.52 ± 5.81	0–50
Sexual Distress (*n* = 1691)	Mean ± SD	Range
FSDS-R	25.84 ± 13.74	0–52
Avoidance of sex (*n* = 1744)	Frequency	Percent
Yes	1177	67.5%
No	567	32.5%
Perceived negative impact on sex (*n* = 1652)	Frequency	Percent
Yes	1327	80.3%
No	325	19.7%
Sexual orientation (*n* = 2057)	Frequency	Percent
Heterosexual	1844	89.6%
Gay/lesbian	47	2.3%
Bisexual	134	6.5%
Other	32	1.6%
Relationship status (*n* = 2058)	Frequency	Percent
Single	381	18.5%
Dating	102	5.0%
Committed relationship	489	23.8%
Married	785	38.1%
De facto	272	13.2%
Divorced	26	1.3%
Widowed	3	0.1%

**Table 2:** Spearman’s Rho for demographic variables, endometriosis symptom frequency, dyspareunia, sexual distress, avoidance of sex, and perceived negative impact of endometriosis symptoms on sex life (perceived negative impact).

	Age	Years with a Diagnosis	Symptom Frequency	Dyspareunia	Sexual Distress	Avoidance of Sex	Perceived Negative Impact on Sex Life
Age	-						
Years with a diagnosis	0.43 **	-					
Symptom frequency	−0.14 **	0.01	-				
Dyspareunia	−0.14 **	−0.04	0.37 **	-			
Sexual distress	−0.12 **	−0.05 *	0.32 **	0.53 **	-		
Avoidance of Sex	−0.01	−0.03	0.28 **	0.46 **	0.50 **	-	
Perceived Negative Impact on Sex Life	0.08 **	−0.01	0.27 **	0.44 **	0.49 **	0.61 **	-

Note: ** Represents correlations significant at *p* ≤ 0.01, * represents correlations significant at *p* ≤ 0.001.

**Table 3:** Results for multivariate logistic regression models examining associations with avoidance of sex and negative impact of endometriosis symptoms on sex life (perceived negative impact).

**Avoidance of Sex**			
**Variables**	**OR** (**95% CI**)	**adjOR** (**95% CI**)	**Sig**
Age	0.99 (0.98–1.01)	1.04 (1.04–1.06)	*p* = 0.001
Years with a diagnosis	0.99 (0.98–1.01)	0.98 (0.95–1.00)	*p* = 0.08
Symptom frequency	1.20 (1.16–1.23)	1.07 (1.03–1.12)	*p* = 0.002
Dyspareunia	3.74 (3.20–4.37)	2.39 (1.99–2.88)	*p* = 0.000
Sexual distress	1.10 (1.09–1.12)	1.08 (1.07–1.09)	*p* = 0.000
Nagelkerke R^2^ = 0.42 for full model			
**Perceived Negative Impact on Sex Life**			
**Variables**	**OR (95% CI)**	**adjOR (95% CI)**	**Sig**
Age	0.97 (0.96–0.99)	1.01 (0.98–1.04)	*p* = 0.37
Years with a diagnosis	0.99 (0.97–1.01)	0.99 (0.96–1.02)	*p* = 0.47
Symptom frequency	1.23 (1.18–1.27)	1.07 (1.02–1.13)	*p* = 0.01
Dyspareunia	4.80 (3.95–5.85)	2.76 (2.18–3.50)	*p* = 0.000
Sexual distress	1.14 (1.12–1.15)	1.11 (1.09–1.13)	*p* = 0.000
Nagelkerke R^2^ = 0.49 for full model			

## Data Availability

The anonymized dataset used during the current study is available from the corresponding author upon reasonable request.

## References

[1] Adamson D., Kennedy S.H., Hummelshoj L. (2010). Creating solutions in endometriosis: Global collaboration through the World Endometriosis Research Foundation. J. Endometr..

[2] Ferrero S., Gillott D.J., Remorgida V., Anserini P., Ragni N., Grudzinskas J.G. (2004). Proteomic Research of New Plasmatic Markers of Endometriosis. Fertil. Steril..

[3] Lemaire G.S. (2004). More Than Just Menstrual Cramps: Symptoms and Uncertainty Among Women with Endometriosis. J. Obstet. Gynecol. Neonatal Nurs..

[4] Peiris S., Chaljub E., Medlock D. (2004). Endometriosis. JAMA.

[5] Bourdel N., Alves J., Pickering G., Ramilo I., Roman H., Canis M. (2015). Systematic review of endometriosis pain assessment: How to choose a scale?. Hum. Reprod. Update.

[6] Stratton P., Berkley K.J. (2010). Chronic pelvic pain and endometriosis: Translational evidence of the relationship and implications. Hum. Reprod. Update.

[7] Heim L.J. (2001). Evaluation and Differential Diagnosis of Dyspareunia. Am. Fam. Physician.

[8] Yang X., Xu X., Lin L., Xu K., Xu M., Ye J., Shen X. (2021). Sexual function in patients with endometriosis: A prospective case-control study in China. J. Int. Med. Res..

[9] Di Donato N.D., Montanari G., Benfenati A., Monti G., Bertoldo V., Mauloni M., Seracchioli R. (2014). Do women with endometriosis have to worry about sex?. Eur. J. Obstet. Gynecol..

[10] Fritzer N., Haas D., Oppelt P., Renner S., Hornung D., Wölfler M., Ulrich U., Fischerlehner G., Sillem M., Hudelist G. (2013). More than Just Bad Sex: Sexual Dysfunction and Distress in Patients with Endometriosis. Eur. J. Obstet. Gynecol..

[11] Cherner R.A., Reissing E.D. (2013). A comparative study of sexual function, behavior, and cognitions of women with lifelong vaginismus. Arch. Sex. Behav..

[12] Laumann E., Paik A., Rosen R. (1999). Sexual Dysfunction in the United States: Prevalence and Predictors. JAMA.

[13] Meana M.M., Binik Y.R., Khalife S., Cohen D. (1997). Biopsychosocial Profile of Women with Dyspareunia. Obstet. Gynecol..

[14] Fritzer N., Tammaa A., Haas D., Oppelt P., Renner S., Hornung D., Wölfler M., Ulrich U., Hudelist G. (2016). When sex is not on fire: A prospective multicentre study evaluating the short-term effects of radical research of endometriosis on quality of sex life and dyspareunia. Eur. J. Obs. Gynecol. Reprod. Biol..

[15] Rush G., Misajon R. (2018). Examining Subjective Wellbeing and Health-related Quality of Life in Women with Endometriosis. Health Care Women Int..

[16] Rush G., Misajon R., Hunter J., Gardner J., O’Brien K. (2019). The relationship between endometriosis-related pelvic pain and symptom frequency, and subjective wellbeing. Health Qual. Life Outcomes.

[17] De Graaff A., D’Hooge T., Dunselman G., Dirksen C., Hummelshoj L., Simeons S., Wullschleger M. (2013). The significant effect of endometriosis on physical, mental and social wellbeing: Results from an international cross-sectional survey. Hum. Reprod..

[18] Shum L.K., Bedaiwy M.A., Allaire C., Williams C., Noga H., Albert A., Yong P.J. (2018). Deep Dyspareunia and Sexual Quality of Life in Women with Endometriosis. Sex. Med..

[19] Denny E., Mann C.H. (2007). Endometriosis-associated Dyspareunia: The Impact on Women’s Lives. J. Fam. Plan. Reprod. Health Care.

[20] Pluchino N., Wenger J.M., Petignat P., Tal R., Bolmont M., Taylor H.S., Bianchi-Demicheli F. (2016). Sexual Function in Endometriosis Patients and Their Partners: Effect of the Disease and Consequences of Treatment. Hum. Reprod. Update.

[21] Bernuit D., Ebert A., Halis G., Strothmann A., Gerlinger C., Geppert K., Faustmann T. (2011). Female perspectives on endometriosis: Findings from the uterine bleeding and pain women’s research study. J. Endometr..

[22] Tabachnick B.G., Fidell L.S. (1996). Using Multivariate Statistics.

[23] Biberoglu K.O., Behrman S.J. (1980). Dosage aspects of danazol therapy in endometriosis: Short term and long-term effectiveness. Am. J. Obs. Gynecol..

[24] Derogatis L.R., Clayton A.H., Goldstein a Lewis-D’Agostino D., Wunderlich G., Cotton D. (2011). E-Diary and Female Sexual Distress Scale in Evaluating Distress in Hypoactive Sexual Desire Disorder. J. Sex Res..

[25] Zarbo C., Brugnera A., Compare A., Secomandi R., Candeloro I., Malandrino C., Frigerio L. (2019). Negative metacognitive beliefs predict sexual distress over and above pain in women with endometriosis. Arch. Women’s Ment. Health.

[26] Jones G., Jenkinson C., Kennedy S. (2004). The Impact of Endometriosis upon Quality of Life: A Qualitative Analysis. J. Psychosom. Obstet. Gynecol..

[27] Fagervold B., Jenssen M., Hummelshoj L., Moen M. (2009). Life after a Diagnosis with Endometriosis—A 15 Years Follow-up Study. Acta Obstet. Gynecol. Scand..

[28] Lethem J., Slade P.D., Troup J.D., Bentley G. (1983). Outline of a Fear-Avoidance Model of exaggerated pain perception—I. Behav. Res. Ther..

[29] Alappattu M.J., Bishop M.D. (2011). Psychological factors in chronic pelvic pain in women: Relevance and application of the fear-avoidance model of pain. Phys. Ther..

[30] Saha R., Marions L., Tornvall P. (2017). Validity of self-reported endometriosis and endometriosis-related questions in a Swedish female twin cohort. Fertil. Steril..

